# Immobilization of peroxidase enzyme onto the porous silicon structure for enhancing its activity and stability

**DOI:** 10.1186/1556-276X-9-409

**Published:** 2014-08-21

**Authors:** Padmavati Sahare, Marcela Ayala, Rafael Vazquez-Duhalt, Vivechana Agrawal

**Affiliations:** 1 Centro de Investigacion en Ingenieria y Ciencias Aplicadas, UAEM, Av. Universidad 1001, Cuernavaca, Morelos 62209, México; 2 Instituto de Biotecnología Universidad Nacional Autónoma de México, Av. Universidad 2001, Cuernavaca, Morelos 62250, México; 3 Centro de Nanociencias y Nanotecnología, Universidad Nacional Autónoma de México, Km 107 Carretera Tijuana-Ensenada, Apdo Postal 14, CP. 22800 Ensenada, B.C., México

**Keywords:** Porous silicon, Peroxidase, Immobilization, Microreactors

## Abstract

In this work, a commercial peroxidase was immobilized onto porous silicon (PS) support functionalized with 3-aminopropyldiethoxysilane (APDES) and the performance of the obtained catalytic microreactor was studied. The immobilization steps were monitored and the activity of the immobilized enzyme in the PS pores was spectrophotometrically determined. The enzyme immobilization in porous silicon has demonstrated its potential as highly efficient enzymatic reactor. The effect of a polar organic solvent (acetonitrile) and the temperature (up to 50°C) on the activity and stability of the biocatalytic microreactor were studied. After 2-h incubation in organic solvent, the microreactor retained 80% of its initial activity in contrast to the system with free soluble peroxidase that lost 95% of its activity in the same period of time. Peroxidase immobilized into the spaces of the porous silicon support would be perspective for applications in treatments for environmental security such as removal of leached dye in textile industry or in treatment of different industrial effluents. The system can be also applied in the field of biomedicine.

## Background

Among microelectronic materials, silicon (Si) has the most mature and low-cost technology; hence, several research groups are approaching Si-compatible technology as an innovative platform for biosensors. Porous silicon has been intensively investigated for a variety of applications such as chemical and biological sensors, medical diagnostics, optical band pass filters, microchemical reactors, and microfuel cells [1]. Moreover, Si-based matrixes have been proved to be a very useful support for the immobilization of enzymes thanks to their capability of retaining biological activity [2]. Silicon (Si) received a lot of attention due to its specific semiconductor properties and furthermore because it allows the development of a broad range of micropatterning processes in order to achieve functional features for future integration in complex systems.

Furthermore, the Si-H and Si-OH groups on porous silicon surface can be easily modified by many reactive reagents and derivatives with receptors, thus enabling the identification of ligands [3]. Microreactors are miniaturized reaction systems fabricated by microtechnology and precision engineering. The microreactors work with micro and nanoliter volumes of reaction media and ensure high efficiency and reproducibility of biocatalytic processes. Enzymatic microreactors have been already proposed as integrated components termed lab-on-a-chip for analytical applications in micrototal analysis systems (MTAS) [4]. There, the immobilization strategies to graft different chemical substances on the surface of a microreactor, a support, are used for a design of necessary conditions within the microreactor spaces. Surface modification by silanization is a very common method for particle functionalization. High density of free amino groups (-NH2) lying outwards the particle surface provides an excellent media for further chemical surface modification such as enzyme cross-linking with glutaraldehyde [5]. The immobilization of enzymes in microreactors is mostly carried out in a covalent way. The main advantage of covalent immobilization is the retention of the enzyme during the whole biocatalytic process [6]. Actually, immobilization is a well-established approach in a wide range of industrial applications. Both synthetic and natural inorganic materials such as clay, glass beads, silice-based materials, and celite have been used to immobilize enzymes, the natural catalysts for many biological processes. Among them, mesoporous silicates are the most interesting due to their attractive properties, availability, and simple preparation [7]. Peroxidase immobilization on inorganic mesoporous silicates has proven to be an interesting alternative to improve enzyme functionality [8]. The large regular repeating structures of photonic porous silicon structure offer the possibility of adsorbing or entrapping large biomolecules within their pores, providing a suitable microenvironment to stabilize the enzyme.

Peroxidases (EC 1.11.1.7, *etc.*) belong to a large family of enzymes that participate in a large number of natural processes developed in living organisms. They are ubiquitous in fungi, plants, and vertebrates [9]. Their principal active sites contain a heme prosthetic group or, alternately, residues of redox-active cysteine or seleno-cysteine groups that are able to oxidize a large number of organic compounds initiated by one electron oxidation step [10]. For all peroxidases, the natural substrate is hydrogen peroxide, but the oxidative process can be performed with many other organic hydro-peroxides such as lipid peroxides. In the oxidation of phenols or aromatic amines, peroxidases produce free radicals that may dimerise or polymerize and thus, in general, form products that are much less soluble in water. This property might be used in removing carcinogenic aromatic amines and phenols from industrial aqueous effluents. Enzymes are also involved in degradation of aromatic compounds and other xenobiotics, including pesticides, polycyclic aromatic hydrocarbons, and dioxins [11], and thus can be used for removal of aromatic pollutants [12,13] as antioxidant [14], as indicators for food processing [15], in bioelectrodes [16] and in the synthesis of conducting materials [17]. Peroxidases could also be used in the synthesis of fine chemicals and optically and biologically active oxide. Despite the obviously practical value of peroxidases, at present, their commercial uses are limited, primarily due to its low stability in the presence of hydrogen peroxide, their natural substrate. All heme-proteins, including peroxidases, are inactivated in the presence of some concentrations of hydrogen peroxide. This process, described as a suicide inactivation, is especially important in the absence of reducing substrates, but its mechanism has not been yet fully elucidated [18]. Although the interest to peroxidase started several decades ago, their application as biocatalysts in industrial processes is still negligible due to its inherent instability under operational conditions, mainly caused by the inactivation in the presence of hydrogen peroxide. The development of techniques for enzyme stabilizing can improve a number of biocatalytic industrial processes. In this work, peroxidase enzyme has been immobilized onto porous silicon (PS) supports for the possible prevention from its self-inactivation and its stability under different operational conditions has been analyzed.

## Methods

A commercial peroxidase, Baylase® RP, was kindly donated by Bayer Mexico (Mexico, Federal District, Mexico). Crystalline silicon was a product from Cemat Silicon (Warsaw, Poland). Glutaraldehyde, 3-aminopropyldiethoxysilane, guaiacol, and bovine serum albumin were from Sigma-Aldrich (St. Louis, MO, USA). Bradford reagent was from Bio-Rad (Hercules, CA, USA). All other chemical reagents used in our experiment were of analytical grade without further purification.

### Microreactor fabrication

Fabrication of porous silicon(PS) <100 > oriented, heavily doped p-type Si wafers with resistivity 0.002 to 0.005 ohm-cm were electrochemically etched with an electrolyte composed of HF/ethanol/glycerol (3:7:1 (*v*/*v*)) at a constant current density of 50 mA cm^-2^ for 170 s to obtain a porous layer of 3,000 ± 60 nm.

### Functionalization of porous support

The porous silicon samples were subjected to thermal oxidation in air at 600°C for 60 min. Silanization process with 3-aminopropyldiethoxysilane (APDES) was performed by immersing the sample in a 5% APDES in toluene for a period of 1 h and annealed at 110°C for 15 min. Glutaraldehyde (GTA, 2.5%) in phosphate buffer pH 6.0 was subsequently coupled to the support for 1 h and finally incubated with peroxidase for 24 h at 4°C. After each step of functionalization, the percent reflectance was measured and the chemical modification of the surface was verified by FTIR.

### RIFTS, SEM, FTIR, and gravimetric measurement of enzymatic microreactor

Reflective interferometric Fourier transform method provides a fast and convenient method of extracting the basic optical parameters modified during the bio-functionalization steps onto of the PS surface. This method presents high sensitivity to small changes in the average refractive index of the porous thin film, allowing for direct and real-time monitoring of the binding of different species to the pore walls [19-22]. Reflective interferometric Fourier transform spectroscopy RIFTS analysis was performed on the specular reflectivity spectra of the PS measured with UV-VIS-NIR spectrophotometer (PerkinElmer Lambda 950, Waltham, MA, USA). As gravimetric measurement is the most direct method of determining the porosity of porous silicon [23-25], the measured porosity of the sample is found to be approximately 80%.

The surface and cross section image of mesoporous silicon was obtained by scanning electron microscope (SEM). Fourier transform infrared (FTIR) spectroscopy was used to identify and characterize the functional groups on the porous silicon surface. The FTIR spectra were collected at a resolution of 2 cm^-1^ on a Cary 640/660 FTIR Spectrometer - with an ATR accessory (Agilent Technologies, Mexico, Federal District, Mexico).

### Enzyme assays

Steady-state measurements for peroxidase activity were carried out spectrophotometrically using guaiacol as electron donor substrate. Peroxidase activity was measured in 1 mL reaction solution containing 60 mM sodium phosphate buffer pH 6.0 at 25 to 28°C using 3 mM guaiacol, 1 mM hydrogen peroxide as the substrates and by monitoring the absorbance changes at *λ* = 470 nm using molar extinction coefficient value of 26.6 mM^-1^ cm^-1^ for the product tetra-guaiacol formed by the enzymatic reaction [26]. One unit of peroxidase activity was defined as the amount of enzyme that caused the formation of micromoles of tetraguaiacol per min. The protein content was determined by Bradford method with the BioRad protein reagent.

### Specific and non-specific immobilization

In an effort to compare the specific and non-specific immobilization of the enzyme load onto the microreactors, three different microreactors has been designed, (1) oxidized support immobilized with enzyme, (2) oxidized and ADPES treated then enzyme immobilization, and (3) oxidized, ADPES, and glutaraldehyde-activated surface incubated with the enzyme. The peroxidase activity of the anchored enzymes onto the pores of microreactors was detected by absorption spectroscopy using guaiacol as substrate at 470 nm.

### Stability assays

Three different stabilities were tested for soluble and immobilized peroxidase preparations: Thermostability by incubating at 50°C, stability to organic solvent by incubating in 50% acetronitrile, and against inactivation in the presence of hydrogen peroxide (1 mM). In all cases, aliquots of each sample were withdrawn at different times and assayed for enzymatic activity under the standard condition. The data were adjusted to first-order rate model in order to calculate inactivation rate constants under each condition.

## Results and discussion

### Preparation of porous silicon substrates

**Figure 1:**
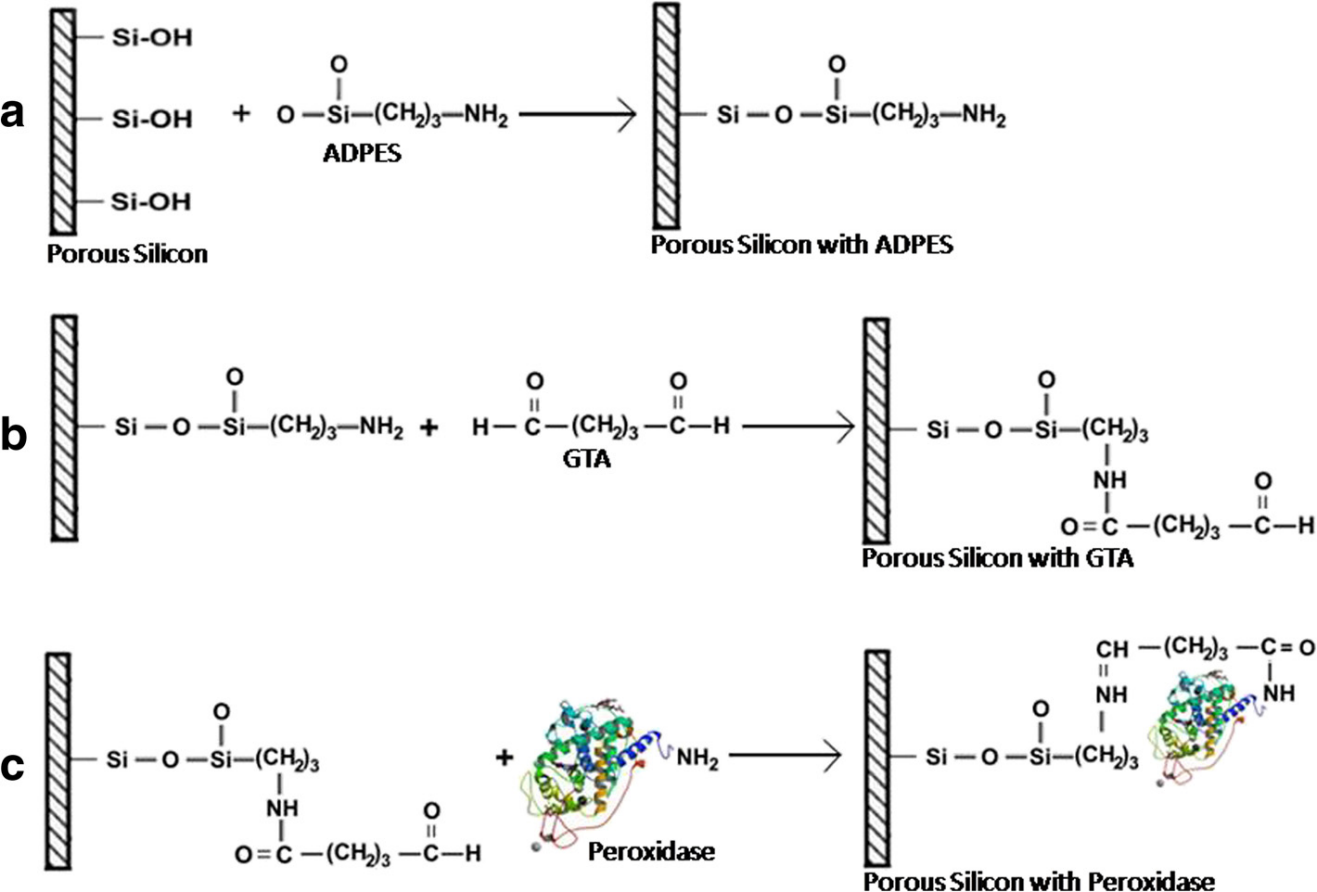
**Schematic diagram illustrating the general process from porous silicon functionalization to enzyme coupling. (a)** Functionalization of oxidized porous support with ADPES. **(b)** Attachment of aldehyde group using glutaraldehyde. **(c)** Covalent attachment of peroxidase to the support through the formation of peptide bond between the aldehyde group and amino acids of the enzyme.

**Figure 2:**
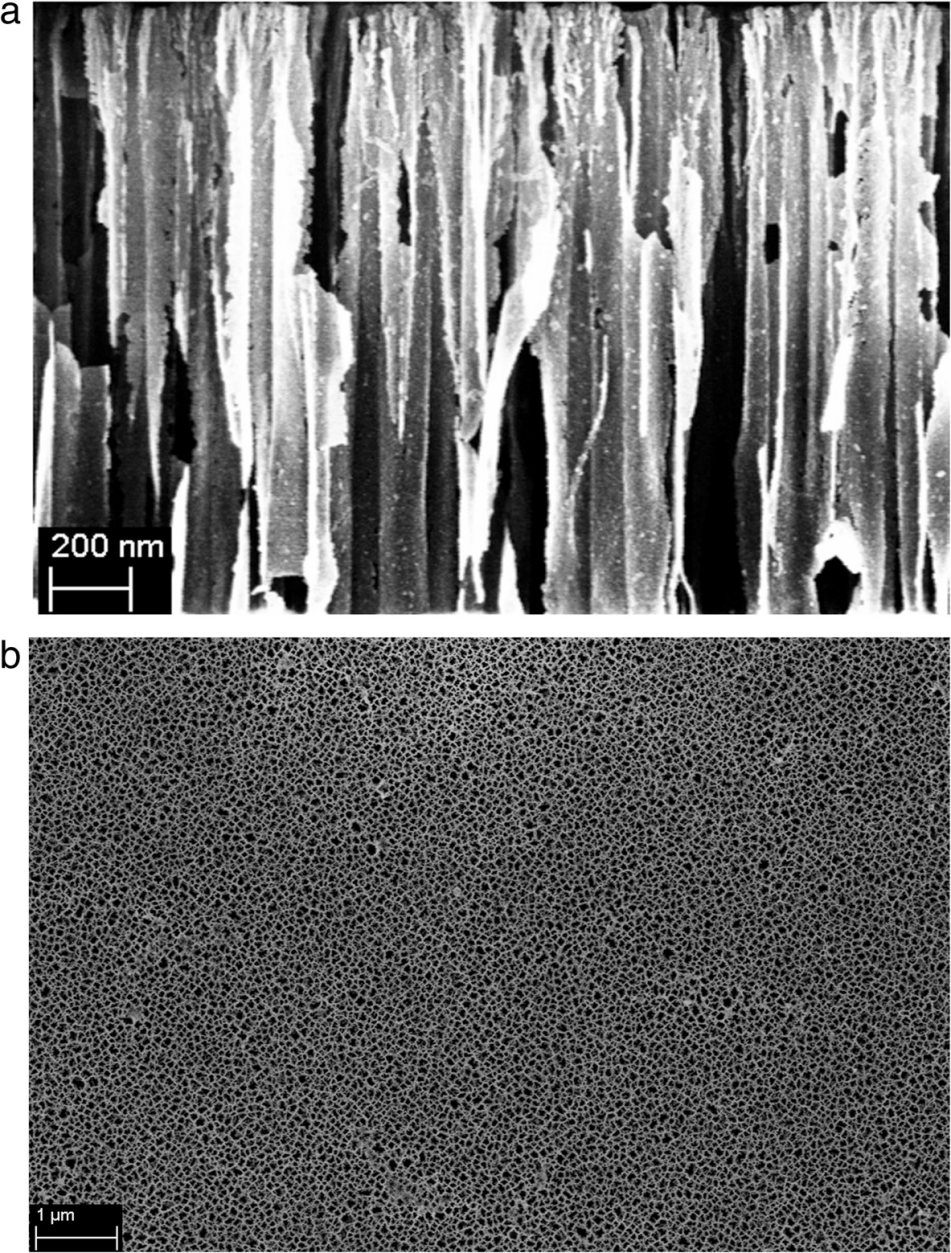
SEM observation of porous silicon structure fabricated, (a) cross section, (b) sample surface.

As shown in Figure  1, the oxidized samples were epoxy-silanized with ADPES to obtain an amine-terminated group. 3-Aminopropyl (diethoxy) methyl silane have been used for surface modification [27], as their bi-functional nature is expected to offer the possibility to covalently attach a bio-molecule, either directly or through a linker. Supports activated with glutaraldehyde or the treatment of the adsorbed enzymes with glutaraldehyde produces a covalent attachment of the enzyme onto the support with glutaraldehyde as a spacer arm, conferring stability to covalently bound enzymes [28]. A detailed view of the surface morphology and thickness has been obtained using the scanning electron microscope (SEM). The porous layer is 3,000 ± 60 nm thick shown in Figure  2a, with interconnecting cylindrical pores ranging in diameter from 30 to 50 nm can be seen in Figure  2b. The pore size distribution is relatively uniform and the columnar walls are thin.

### Reflective interferometric Fourier transform spectroscopy

**Figure 3:**
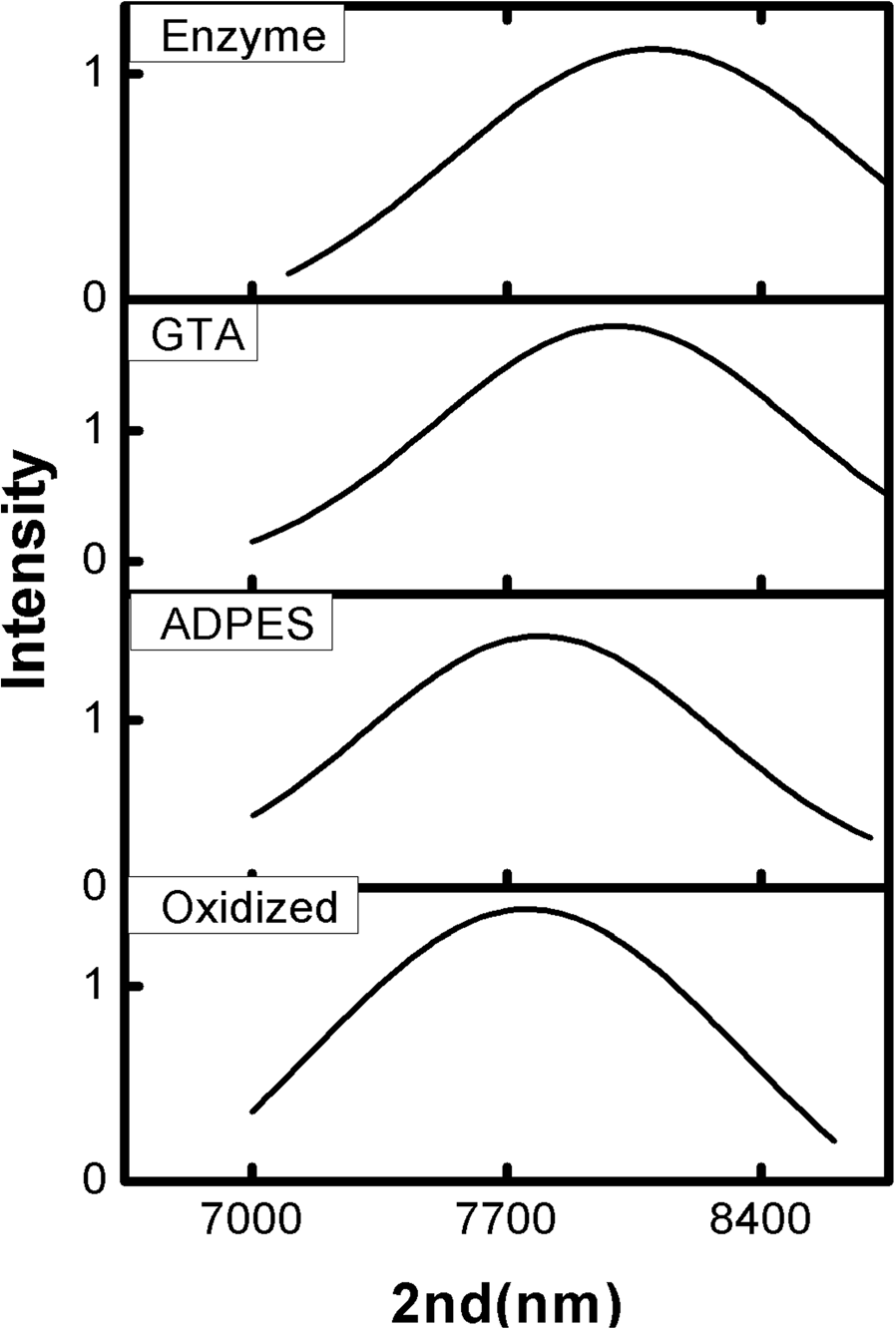
**Shift in optical thickness (2nd) of the porous silicon structure after functionalization.** The increase of the refractive index after the incubation in APDES and GTA results in a red shift in the reflectance peak, and hence, the corresponding change in optical thickness is observed.

Fourier transform are widely involved in spectroscopy in all research areas that require high accuracy, sensitivity, and resolution [29-31]. It should be noted that the nanostructure is designed to allow proper infiltration of the peroxidase enzyme (approximate size of 40 KDa), characterized by an average diameter of 60 to 80 Å, considering a globular conformation The functionalization of each compound was monitored through shift in reflectance peak. It is expected that the chemical modification of the porous nanostructure (as outlined in Figure  3) will result in an increase of the optical thickness (i.e., red shift of second) due to the increase in the average refractive index upon attachment of different species to the pore walls.

### FTIR studies

**Figure 4:**
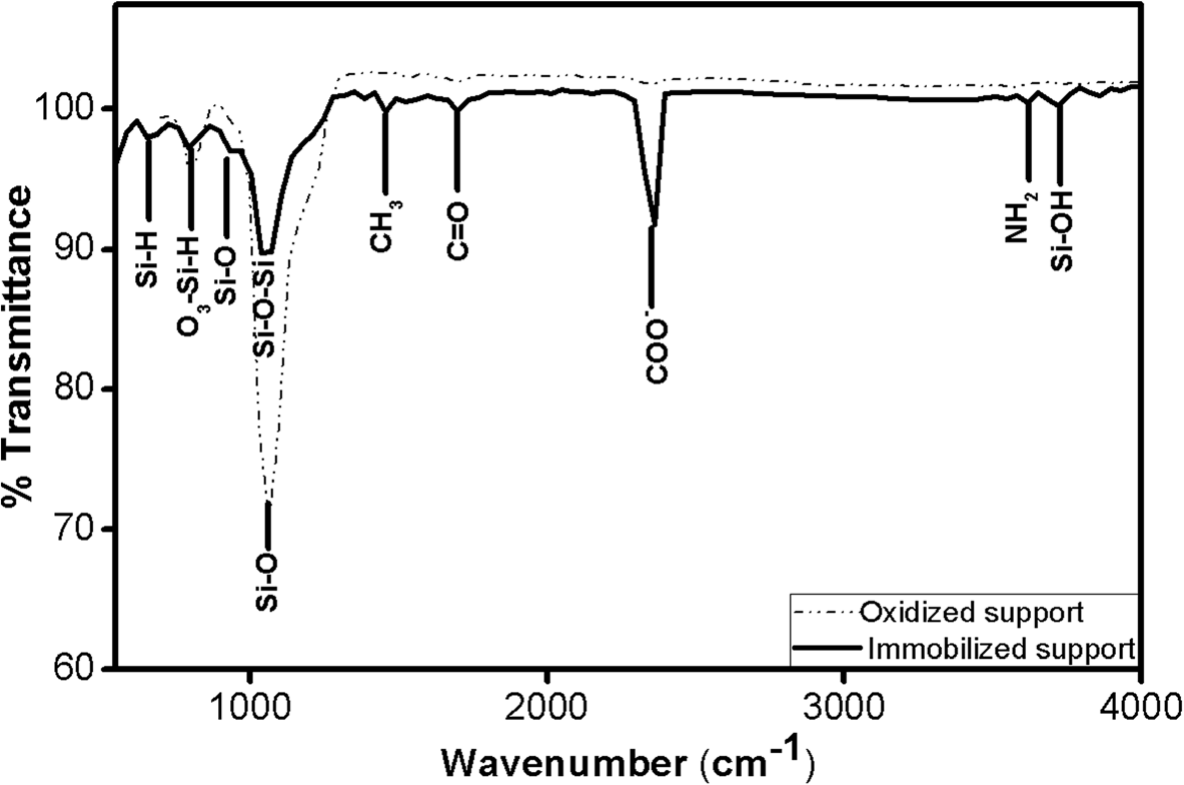
**Attenuated total reflectance (ATR) spectrum of PS structure with immobilized peroxidase taken after all the functionalization steps.** FTIR analysis reveals some characteristic peaks of different functional group and peroxidase that has been infiltrated into the porous support.

Figure  4 shows a FTIR spectrum measured after oxidation step and after immobilization. The reference spectrum of oxidized porous silicon support shows two bands corresponding to the characteristic asymmetric stretching mode of Si-O at 1,050 to 1,100 cm^-1^ and the Si-OH bond at 825 cm^-1^[32]. The spectra of immobilized support show a sharp band of silanol at about 3,730 cm^-1^ and a band at 3,350 cm^-1^ correspond to the asymmetric stretching modes of -NH_2_ groups. [33]. Functionalization with ADPES resulted in a band related to Si-O-Si at 1,034 cm^-1^, which confirms that the siloxane bonding between ADPES and oxidized support has taken place [34]. The asymmetric and symmetric deformation modes of the CH3 group from ethoxy moieties of APTES are observed around 1,440 cm^-1^[35]. The peak at 1,691 cm^-1^ corresponds to Amide I, the most intense absorption band in proteins. It is primarily governed by the stretching vibrations of the C = O (70 to 85%) and C-N groups (10 to 20%) [36]. The setup of spectroscopic analysis presented above confirms the effective immobilization of a biocatalyst onto the surface of PS support.

### Specific and non-specific immobilization

Table  1 shows the enzyme activity and protein load of three different microreactors. The microreactor in which enzyme was loaded after glutaraldehyde shows maximum activity in comparison to the other two microreactors. Type of activation, its presence, distribution, and density of functional groups determines the activity yields of an immobilization reaction and operational stability of the carrier-fixed enzyme. Compared to non-specific adsorption, specific adsorption often orients the enzyme molecule in a direction allowed by the nature of binding and the spatial complementary effect which may contribute for the higher activity in glutaraldehyde-activated microreactors.

**Table 1:** Effect of immobilization chemistry on the enzyme loading onto PS support

**Microreactors**	**Enzyme activity (U)**	**Protein (mg)**
Oxidized + enzyme	0.193/50 ml	1.8/50 ml
Oxidized + ADPES + enzyme	0.276/100 ml	2.4/100 ml
Oxidized + ADPES + GTA + enzyme	0.712/100 ml	3.9/100 ml

### Effect of PS layer thickness on the enzymatic activity

Peroxidase immobilization onto the microreactor with different thickness of the layer indicates that large amount of enzyme has been immobilized onto the thicker layer but are not available for the substrate conversion (data shown in Table  2). In most cases, a large surface area and high porosity are desirable, so that enzyme and substrate (guaiacol) can easily penetrate. A pore size of >30 nm seems to make the internal surface accessible for immobilization of most enzymes. All reactions of immobilized enzymes must obey the physicochemical laws of mass transfer and their interplay with enzyme catalysis [37].

**Table 2:** Effect of PS layer thickness (Si wafer) on the enzymatic activity

**Thickness of the porous layer**	**Enzyme activity**	**Protein**
	**(U cm**^**-2**^**)**	**(mg cm**^**-2**^**)**
Crystalline silicon	No detectable activity	0.32
500 nm	0.576	2.15
4,000 nm	0.456	3.52

### Thermal stability of immobilized peroxidase enzyme

**Figure 5:**
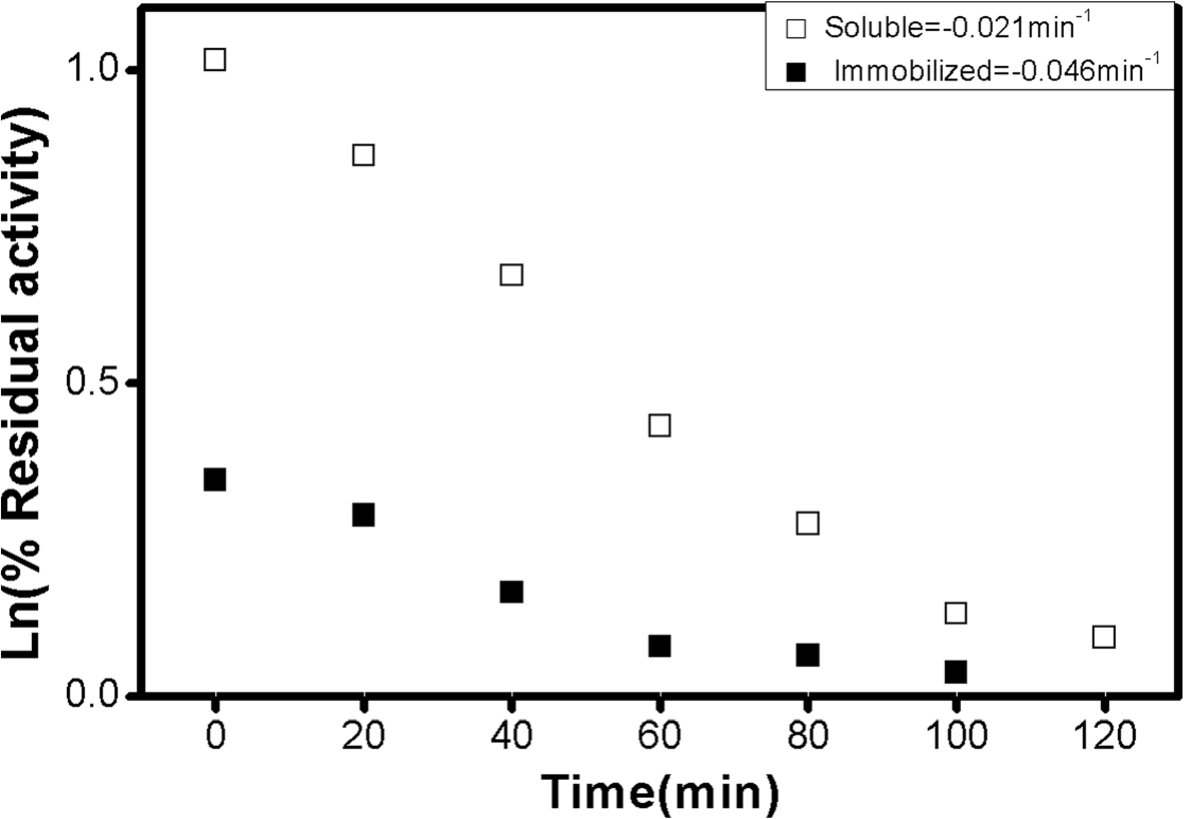
First-order rate constant calculations from semi-logarithmic plot of residual activity of soluble and immobilized peroxidase during incubation (50°C).

Thermo-stability is the ability of an enzyme to resist against thermal unfolding in the absence of substrates. The relative thermal stability of the free *versus* immobilized enzymes was compared at 50°C (Figure 5). The results suggested that the immobilized enzyme is inactivated more rapidly compared to the soluble enzyme as indicated by the inactivation constant rate. The decrease in the thermal stability of the immobilized support is attributed to the thermal conductance of silicon resulting in the major heat transfer from Si support to the enzyme (thermal conductivity of silica 8 W m^**-1**^ k), as has been observed in other reports [38].

### Stability of peroxidase in aqueous-organic solvent mixture

**Figure 6:**
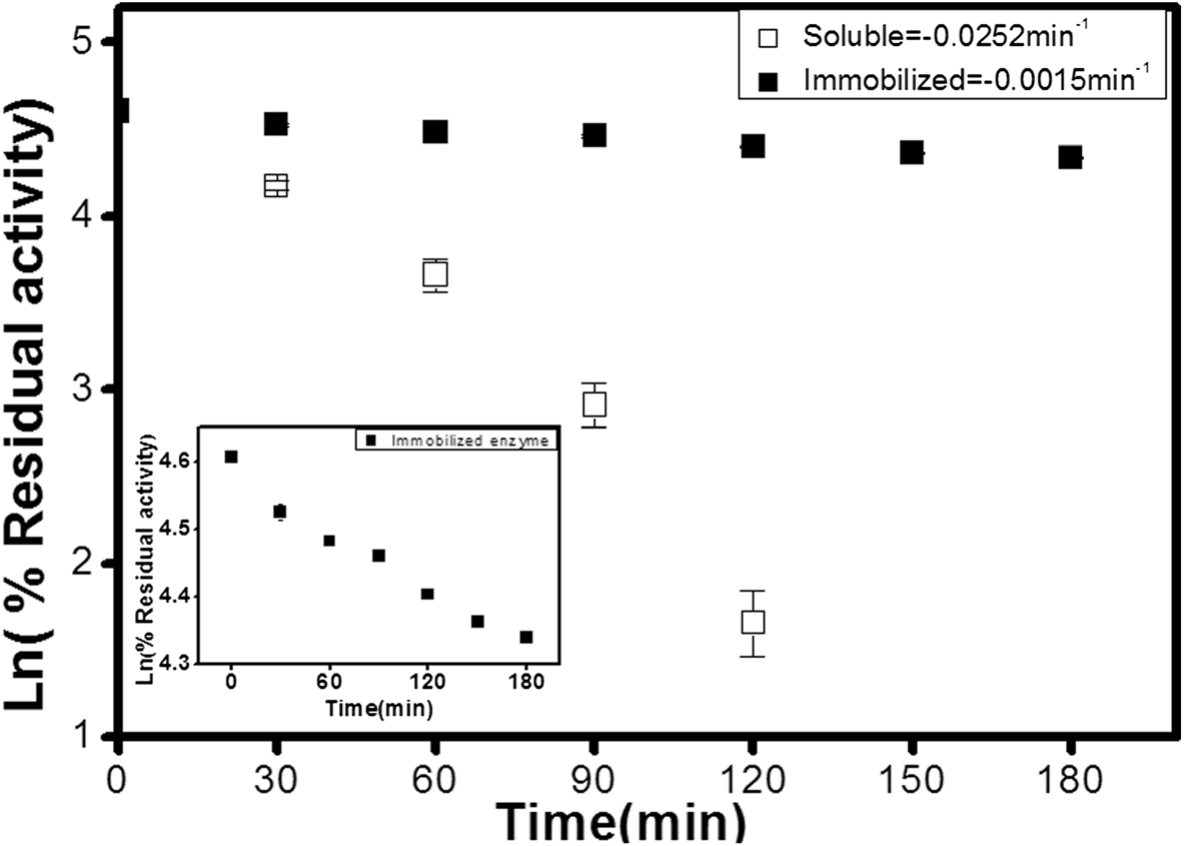
**First-order rate constant calculations from semi-logarithmic plot of residual activity of soluble and immobilized peroxidase during incubation (50% acetonitrile).** The insert shows an amplification of immobilized enzyme profile.

As the stabilization of enzymes is one of the most complex challenges in protein chemistry, the stability of soluble and immobilized peroxidase has also been investigated in aqueous solution containing 50% acetonitrile. As shown in Figure  6, the immobilized peroxidase showed a greater tolerance to acetonitrile by retaining 80% of the catalytic efficiency in comparison to the soluble enzyme which lost 95% of its activity after 2 h. Organic solvents can inactivate enzymes in several ways: the organic solvent molecules can interact with the biocatalyst, disrupting the secondary bonds in the native structure; they can strip the essential water molecules from the hydration shell altering the structure of the enzyme; or they can interact with the active site of the biocatalyst, causing inactivation.

### Stability of peroxidase in the presence of hydrogen peroxide

**Figure 7:**
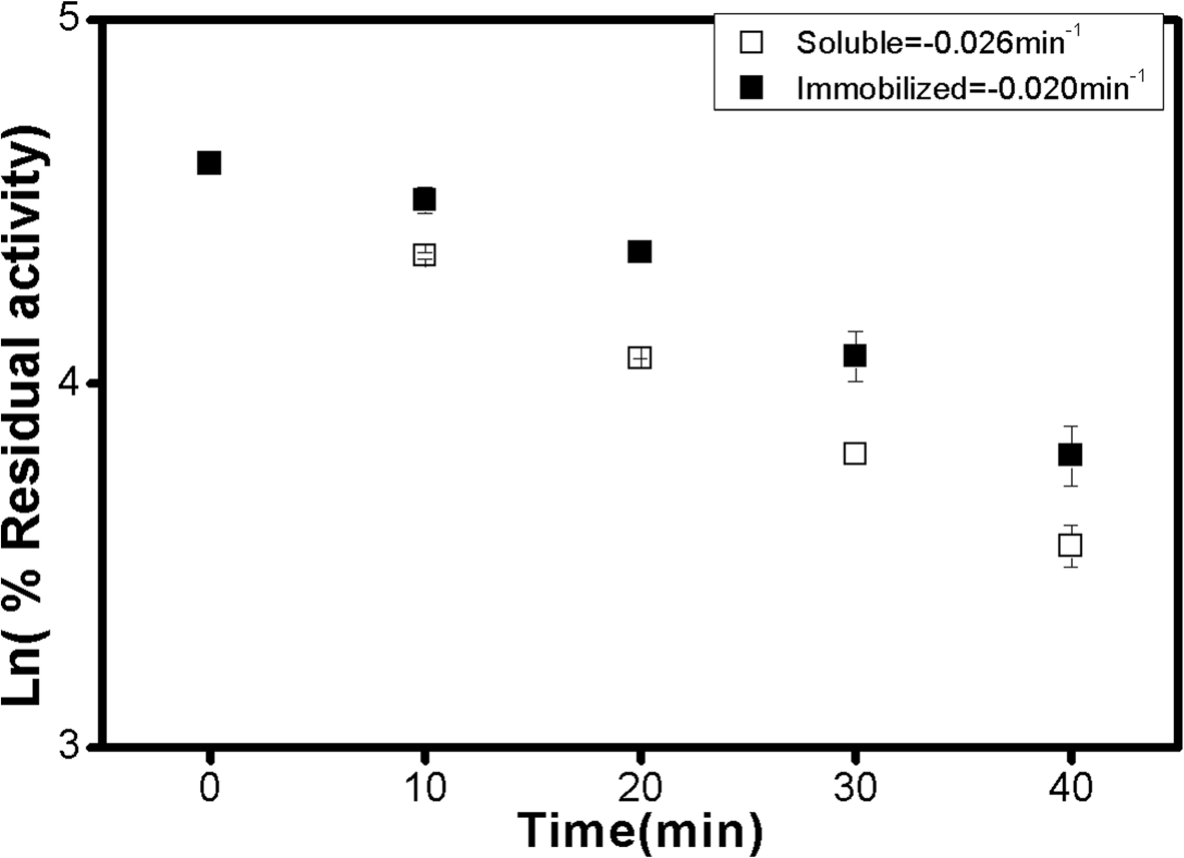
**First-order rate constant calculations from semi-logarithmic plot of residual activity of soluble and immobilized peroxidase with H**_**2**_**O**_**2 **_**incubation.**

The stability of peroxidase in the presence of hydrogen peroxide is a key issue because peroxidase becomes inactive in the presence of excess hydrogen peroxide; therefore, the effects of hydrogen peroxide on the stability of the enzyme were investigated. As expected, the activities of the free peroxidase decreased rapidly in the presence of hydrogen peroxide, with a decrease to less than 50% of the initial activities occurring within 40 min. On the other hand, immobilized peroxidase showed a slightly lower inactivation rate, suggesting no significant protection of the enzyme against hydrogen peroxide, due to the binding of the enzyme to PS matrix as shown in Figure  7.

## Conclusions

This work is focused on porous silicon surface functionalization through the covalent attachment of the peroxidase enzyme with the PS support. The immobilization of the enzyme onto the porous silicon support has been confirmed from the RIFTS and FTIR studies. The study of thickness of the porous layer onto the availability of enzyme showed that higher thickness hinders the passage of substrate into the pores, which results in lower activity. The immobilized support showed lower thermo-stability with respect to soluble/free enzyme due to the major heat transfer through silicon support. The inactivation profile of peroxidase in the presence of acetonitrile indicates that the immobilized peroxidase is protected from acetonitrile deactivation; thus, acetonitrile has been revealed to be a very promising solvent to perform biocatalysis with peroxidase in organic media. While the deactivation of the enzyme in the presence of H_2_O_2_ in immobilized support is almost similar as compared to the soluble enzyme, these results conclude that a commercial peroxidase enzyme immobilized onto the porous silicon nanostructure confers more stability against organic solvents for potential industrial applications.

## Competing interests

The authors declare that they have no competing interests.

## Authors' contributions

P.S. carried out all the experimental work. M.A. helped in the biological part of the experiments. P.S. and V.A. jointly discussed and wrote the manuscript. V.A. and R.V.D. conceived the experiments. All the authors analyzed and discussed the results. All authors read and approved the final manuscript.

## Authors' information

P.S. is a third year PG student at CIICAp, UAEM. RVD is a senior scientist in Biotechnology Institute (IBT) of National Autonomous University of Mexico (UNAM) working in the field of nano-biotechnology and bio-catalysis. MA is a scientist in IBT UNAM. VA is a senior scientist working in Research Centre for Engineering and Applied Sciences in the field of porous silicon and its applications.
